# Exploring the Patient Experience in Behçet’s Syndrome: A Two-Phase Qualitative Study Using a Biographical-Narrative Approach

**DOI:** 10.7759/cureus.88891

**Published:** 2025-07-28

**Authors:** Ariadna Rodríguez-Sánchez, Claudio-Alberto Rodríguez-Suárez, Candelaria de la Merced Díaz-González, Héctor González-de la Torre

**Affiliations:** 1 Nursing, Complejo Hospitalario Universitario Insular Materno Infantil, Las Palmas de Gran Canaria, ESP; 2 Nursing, Universidad de Las Palmas de Gran Canaria, Las Palmas de Gran Canaria, ESP

**Keywords:** autoimmune diseases, behçet syndrome, biographies as topic, qualitative research, review literature as topic

## Abstract

Background

Behçet’s syndrome (BS) is a systemic autoimmune disease, classified among the vasculitides, with an unknown etiology. It significantly impacts the quality of life of those affected. Healthcare professionals must understand the characteristics of this syndrome to provide comprehensive care.

Objectives

The main objective of this study is to synthesize qualitative evidence on BS, and explore the life experiences of a person living with this condition, through a biographical-narrative account to inform clinical and psychosocial care strategies.

Methods

A two-phase study was conducted. Phase 1 involved a qualitative systematic review, including studies in English and Spanish, without restrictions on publication year. A search was conducted in the Medline, Web of Science, and Scopus databases. Themes and sub-themes were extracted to develop the interview guide. In Phase 2, a semi-structured interview was conducted with a woman diagnosed with BS, employing a biographical-narrative design. The interview was recorded, transcribed, and coded, and themes and sub-themes were identified using ATLAS.ti software (version 25.0.1; ATLAS.ti Scientific Software Development GmbH, Berlin, Germany).

Results

The themes (n = 3) and sub-themes (n = 7) identified in the interview were: coping and adaptation strategies (self-care and seeking support), interactions with the healthcare system (medical care and health education), and the experience of living with the disease (emotional well-being, physical discomfort, and vulnerability).

Conclusions

Qualitative literature on BS is limited and focused on adults, revealing gaps in care and psychosocial support. The testimonial collected highlights the everyday barriers faced by individuals with BS, and identifies key areas for clinical and psychosocial intervention. Further multidisciplinary qualitative research is recommended to improve diagnosis, treatment, and comprehensive support.

## Introduction

Globally, more than 10,000 rare diseases have been identified, affecting a small portion of the population. These conditions are often serious and frequently degenerative, with a prevalence of fewer than 5 individuals per 10,000. Rare diseases significantly impact the quality of life of those affected, due to a wide array of chronic and debilitating symptoms that can lead to anxiety, depression, difficulties in work and education, and even social isolation. Moreover, the high cost of treatments and delays in diagnosis impose a considerable economic burden, often extending to the patient’s family and social environment [1].

Behçet’s disease, or Behçet’s syndrome (BS), is classified as a rare disease, characterized by low prevalence, delayed diagnosis, lack of curative treatment, and emotional toll. It is a systemic autoimmune disease, categorized under vasculitis (chronic inflammatory vasculitis) of unknown cause [2,3]. It was first described in 1937 by Turkish dermatologist Hulusi Behçet, who defined the condition as a triad of symptoms: aphthous stomatitis, genital ulcers, and recurrent uveitis [2,3]. According to the International Study Group (ISG), the primary diagnostic criterion is the presence of recurrent oral ulcers occurring at least three times per year [4]. However, beyond oral ulcers, patients may also present with genital ulcers, cutaneous and ocular lesions, joint pain, and, in some cases, vascular, gastrointestinal, or neurological involvement. Although it can occur anywhere in the world, BS is most prevalent in the Silk Road region - which includes Mediterranean, Middle Eastern, and Far Eastern countries. Turkey reports the highest prevalence (20 to 420 cases per 100,000 inhabitants), followed by Iran (80 to 1,000 per 100,000) [5]. In northern Spain, the estimated prevalence is 10.14 per 100,000 people [6].

This disease is associated with numerous challenges, beginning with a complex diagnostic process and progressing through a painful and unpredictable course. These challenges are compounded by a general lack of awareness among healthcare professionals. This scenario has a negative impact on patients’ quality of life, affecting their physical, emotional, and social well-being. Everyday activities, such as exercising, going to the beach, or even eating, can be disrupted by joint pain, genital ulcers, or the fear of flare-ups - all of which also affect interpersonal relationships and work life [2]. For these reasons, conducting qualitative research on BS is crucial to understand the perspectives of those living with the condition and to develop better support strategies [6].

Based on the above, the guiding research question for this study was: What are the lived experiences and daily life challenges of individuals with BS? To answer this, the study aimed to synthesize qualitative evidence on BS and explore the life experiences of a person living with this condition through a biographical-narrative account to inform clinical and psychosocial care strategies.

## Materials and methods

A two-phase methodology was employed. In Phase 1, a review of qualitative studies focusing on the experiences of individuals with BS was conducted. In Phase 2, the life history and personal experiences of a woman living with BS were explored in depth, using the themes identified in the review as a basis.

Phase 1: Review of qualitative studies

Design

A qualitative review was conducted following the methodology proposed by the Joanna Briggs Institute (JBI) [7]. The reporting of results adhered to the Enhancing Transparency in Reporting the Synthesis of Qualitative Research (ENTREQ) guidelines [8]. The research question followed the experiential PICo framework, where the Population (P) refers to individuals with BS, the Phenomenon of Interest (I) to experiences and lived narratives, and the Context (Co) to activities of daily living. The review protocol was not registered.

Information Sources and Search Strategy

The selected databases for data retrieval were Medline (via PubMed), Web of Science (WOS) (via WOS Complete), and Scopus (via Scopus-Elsevier). The search strategy used the MeSH descriptors “Behçet Syndrome” and “Qualitative Research,” as well as the free-text terms “Behçet Disease” and “Behçet” in the title and abstract fields. Boolean operators AND and OR were applied to combine search terms. The searches were conducted by one of the researchers (ARS) between October and November 2024, and were reviewed by another researcher (CARS) using the PRISMA-S framework [9]. An example of the search strategy applied in Medline (October 31, 2024) is as follows: ((“Behçet Syndrome” [Mesh] OR “Behçet Disease”) OR (“Behçet”)) AND (“Qualitative Research” [Mesh])).

Eligibility Criteria

Studies were included if they explored the lived experiences and daily life of individuals diagnosed with BS and used any qualitative methodology, such as grounded theory, phenomenology, or ethnography. Articles in English or Spanish were eligible, with no restriction on publication date. Quantitative studies, literature reviews, non-research publications, and gray literature were excluded.

Screening Process

The retrieved records were exported into an Excel® spreadsheet (Microsoft® Corp., Redmond, WA, USA) for the selection process. After removing duplicates, two researchers (ARS and CARS) screened titles and abstracts. Discrepancies were resolved by a third researcher (HGdlT). Records that passed this initial screening phase were then subjected to full-text review. The full-text screening was conducted independently and blinded by two researchers (ARS and CMDG). Any disagreements were resolved by a third researcher (HGdlT).

To assess the quality of the included studies, the JBI Critical Appraisal Checklist for Qualitative Research was used. A score of 70% or higher (at least 7 out of the 10 items) was considered indicative of good quality. A pilot appraisal was conducted using one study to ensure the adequacy of the screening process.

Data Extraction Process

This process was performed independently and blinded by two researchers (ARS and CARS), with discrepancies resolved by a third researcher (HGdlT). Extracted information included sociodemographic data of the studies (authors, year of publication, and country), participant characteristics (number, gender, age, disease duration, among others), methodological approach (grounded theory, phenomenology, and ethnography), data collection techniques (interview duration, number of focus group participants, among others), and details on identified themes and subthemes.

Phase 2: Life history

Design

In the second phase, a qualitative biographical-narrative design was employed, using the life history of an individual diagnosed with BS. The methodological approach followed Van Manen’s framework [10], which integrates Husserl’s descriptive perspective and Heidegger’s interpretative hermeneutics [11]. Personal experiences with the disease were explored through the participant’s life narrative, which, rather than simply listing isolated events, connects individual episodes with the biographical context and addresses specific phenomena or themes to give meaning to lived experience [12]. The study was conducted at the Universidad de Las Palmas de Gran Canaria (ULPGC), Las Palmas de Gran Canaria, Spain, from October 31, 2024, to March 31, 2025.

Participant and Context

The participant was a 21-year-old woman residing in the Canary Islands, Spain. She began experiencing symptoms at age 11 and was diagnosed with BS at 13. Since then, she has dealt with frequent symptoms such as oral and genital ulcers, joint inflammation, and headaches. Over the course of her illness, she has received various treatments, including corticosteroids, immunosuppressants, and biologics. She is currently being treated with the biological drug etanercept, which has provided her with greater stability than previous therapies. However, none of the treatments has led to complete, long-term remission.

Throughout the years, she has been seen by multiple medical specialties - family physicians, rheumatologists, and dermatologists - within both public and private healthcare systems, the latter sought due to delays and lack of clarity in care. She currently receives care at the Hospital Insular de Las Palmas de Gran Canaria and the El Calero Health Center (Canary Islands, Spain), where she feels greater engagement from the healthcare staff.

She is currently pursuing a higher education program in clinical laboratory science. She has a stable relationship with her partner but lives with her family, which includes her mother, father, and brother. Although she has never received psychological support, she acknowledges its importance and expresses interest in accessing such services. The illness has had a profound impact on her personal, family, and social life, affecting her daily routines, education, and social interactions. Despite these challenges, she demonstrates resilience and a strong determination to adapt, understand her body, and maintain a positive mindset in facing the disease.

Research Process

Data were collected through a semi-structured, in-person interview conducted privately without the presence of others. The participant was previously known to one of the researchers (ARS) and agreed to share her life history upon request. The interview was conducted on March 17, 2025, by researcher ARS, in a comfortable, private setting without interruptions. No other individuals or researchers were present.

Sociodemographic variables (e.g., age, sex, disease duration, and educational background) were collected at the outset. The themes and sub-themes identified during the review phase informed the development of the interview questionnaire. The complete interview guide is presented as an interview script in Table 1.

**Table 1:** Interview script

Question number	Content of the question
Question 1	Could you tell me about the disease and what your life was like before you were diagnosed?
Question 2	How do you remember the moment you were told you had Behçet's syndrome?
Question 3	What are the symptoms you experience most frequently and how do those symptoms impact your daily life?
Question 4	What strategies do you use to manage symptoms?
Question 5	Have health professionals provided you with information on outbreak prevention? What types of recommendations have they given you?
Question 6	What changes have you made in your routine for the improvement of the disease?
Question 7	Do you feel understood by healthcare professionals and do you feel comfortable contacting them in case of questions or outbreaks?
Question 8	Have you ever experienced a situation in the healthcare setting in which you have felt violated or uncomfortable?
Question 9	What treatment are you currently undergoing?
Question 10	Have you been provided with information to understand how the treatment works?
Question 11	Do you have hope that they will find an effective treatment or cure for the disease?
Question 12	How does the disease affect you psychologically? What emotions are most present when dealing with your disease?
Question 13	Could you share strategies that have helped you cope with the disease?
Question 14	How has the disease affected your social life in general?
Question 15	How has your family experienced the disease and do you feel supported by your family?
Question 16	Are you in a dating relationship? How has the disease affected your social relationships?
Question 17	How has the disease affected your sex life?
Question 18	Do you know of cases of people in the same situation as you? How have you encountered them?
Question 19	Do you feel comfortable sharing your Behçet's problems with the people close to you?
Question 20	What do you do? Do you work or study?
Question 21	How has the disease affected your work life or your studies?
Question 22	Do you feel motivated to perform activities in your daily life? Do you have dreams and aspirations?
Question 23	What do you hope for the future regarding your life with Behçet's syndrome?
Question 24	Would you like to share more about your experience?

Analysis Process

The interview was audio-recorded using an iPhone 13® voice recorder app (Apple, Inc., Cupertino, CA, USA) and later transcribed into text using Microsoft Word® (Microsoft® Corp., Redmond, WA, USA). Transcription was performed by the same researcher (ARS), and then shared with the participant, who reviewed and approved the content.

An inductive abstraction process was used for data analysis. Verbatims were analyzed to identify units of meaning (UoMs) or codes - segments that conveyed recurring patterns with similar meanings. These UoMs were then grouped into sub-themes and overarching themes, aligned with the categories identified in Phase 1. The emerging sub-themes and themes from the interview were subsequently compared with those identified in the literature review to recognize patterns consistent with findings reported in previous studies. 

Additionally, conceptual elements from Riegel et al.’s Middle-Range Theory of Self-Care were incorporated into the coding process [13]. This theory describes how individuals with chronic conditions make decisions to maintain health and respond to symptoms within a self-care framework, which includes three dimensions: self-care maintenance, self-care monitoring, and self-care management. The model also considers personal and contextual factors such as knowledge, previous experience, self-efficacy, cognitive function, social support, values, preferences, and access to resources. Two key components - decision-making and self-care outcomes - were also considered.

The analysis was conducted using ATLAS.ti software (version 25.0.1; ATLAS.ti Scientific Software Development GmbH, Berlin, Germany).

Rigor and Reflexivity

Lincoln and Guba’s criteria for ensuring rigor in qualitative research were followed [14]. Credibility was ensured through detailed data collection in all phases and accurate transcription of the interview. The participant also verified the findings to ensure the interpretations accurately reflected her experience, strengthening the validity of the life history. Coding was performed collaboratively: an initial analysis by the principal investigator was followed by triangulation with the research advisor to reach a consensus on identified subthemes and themes, ensuring interpretive coherence. Transferability was enhanced by providing a rich description of the setting, participant, context, and methods. Dependability was evaluated through external review by two independent researchers (CMDG and HGdlT), who were not involved in data collection or analysis. Confirmability was established through triangulation between the transcript and findings from the Phase 1 review, and through joint review by two researchers (ARS and CARS), supporting coherence and analytical depth. The reporting of results adhered to the Consolidated Criteria for Reporting Qualitative Research (COREQ) guidelines [15].

Ethical considerations

The study was approved by the Research Ethics Committee of the Hospital Universitario de Gran Canaria Doctor Negrín, Las Palmas, Spain, under approval number 2025-018-1. Prior to the interview, the participant was provided with and informed about the consent form, data protection procedures, and the right to withdraw. The consent form was signed before proceeding.

## Results

The life history yielded 40 coded quotes (verbatims), which were assigned to 114 codes. These codes were grouped into seven analytical categories (sub-themes) and three main themes. Themes and sub-themes identified in the interview are presented in Table 2.

**Table 2:** Themes and sub-themes identified in the interview

Themes	Sub-themes
Coping and Adaptation Strategies	Self-care
	Seeking support
Relationship With the Healthcare System	Medical care
	Health education
Living With the Disease	Emotional well-being
	Physical discomfort
	Vulnerability

Coping and adaptation strategies

The participant emphasized the importance of developing personal strategies for coping and adaptation.

Self-Care

Self-care emerged as a central element for maintaining a healthy lifestyle, as illustrated in the following verbatim:

“To avoid ulcers, I try not to eat acidic foods, juices, chocolate-things I’ve learned don’t sit well with me. I’ve always been told to exercise, rest, and use creams that relieve the pain.”

However, she acknowledged that this approach was less consistent during adolescence:

“When I was a teenager, I didn’t care much, but after seeing the disease getting worse, I started taking it seriously. I’ve tried to adapt by changing small habits, having willpower, and staying positive.”

Seeking Support

Support from family, a partner, and friends was another key pillar:

“…I’ve always had close friends who support me. I’ve never felt like a freak. I’ve never felt judged-they really empathize with my situation.”

“My mom was very concerned and supported me a lot with sports, but she set limits so I wouldn’t overexert myself.”

“I have an understanding partner.”

Relationship with the healthcare system

The participant discussed her experiences with the healthcare system, emphasizing the need to feel understood and supported by healthcare professionals and the importance of information to manage her illness.

Medical Care

She spoke of mixed experiences with care providers. Some were sources of support:

“…since I was diagnosed by the rheumatologist, I’ve had a very good relationship with him. He makes me feel comfortable and understood. My family doctor is also very interested in my case.”

Other experiences were negative:

“When I go to the emergency room, I’ve waited for hours only to be told they don’t know what to do.”

“…I had lots of mouth ulcers, and the nurse just kept telling me to speak properly.”

Health Education

The participant noted a lack of specific information on how to manage her illness, beyond general advice:

“…they’ve told me to avoid stressful situations, but not much else. I’ve had to figure things out on my own by looking for recommendations and strategies online.”

She also mentioned never receiving guidance about the impact of the disease on her sexual life:

“That’s what has affected my relationship the most.”

Additionally, she explained that she sometimes preferred not to know about treatment side effects to avoid anxiety:

“…I’d rather not be told the side effects, because I get paranoid and feel worse.”

She described the various treatments she had tried, highlighting improvement with the current drug:

“Three months ago I started Etanercept, and I inject it at home every two weeks... so far, I feel much better with this one, and I hope it stays that way.”

Living with the disease

The participant described her life with BS as an emotional rollercoaster marked by ongoing changes.

Emotional Well-Being

She described a shift from frustration and uncertainty to greater acceptance, accompanied by moments of hope, personal adaptation, and reflection on the future:

“I used to get frustrated because I didn’t understand why I had this disease, but now I’ve adapted and face it with more strength. Right now I feel very hopeful because the treatment is working…”

She emphasized adolescence as one of the most difficult periods in her life:

“…when I played basketball, it affected my social life; I watched my teammates train and go through the entire session…”

“In high school, being in class and not being able to speak because of mouth ulcers, or feeling dizzy from so much corticosteroid medication, made me feel uncomfortable.”

Physical Discomfort

Chronic pain had a significant impact on her daily life, affecting eating, academic activities, work, and leisure. She had to develop adaptation strategies:

“The symptoms I experience most often are ulcers and joint pain. Going to the bathroom is painful because the genital ulcers sting.”

“My joints used to only limit me during exercise, but now they also affect my ability to keep up with work.”

“When I have a flare-up, it’s like I’ve never exercised in my life.”

“I learned to set limits to avoid exhaustion and ending up with a flare.”

“Studying affects me… the day after an exam, I wake up with more symptoms.”

Vulnerability

Regarding the sense of vulnerability that accompanies the disease, she shared experiences marked by fear, loneliness, and emotional insecurity about the future:

“When I was diagnosed, it was awful-I was 12, in a cold consultation room, and my parents weren’t there. At first, I was scared because I didn’t know if I’d be able to live with the disease.”

She also reflected on the burden of medication:

“…I feel fear and uncertainty - it’s a harsh treatment, they’re immunosuppressants, and I know it’s not good to be on them all my life… when a treatment fails, I feel sad, angry, and powerless.”

“…no matter how much willpower or motivation I have, I don’t see progress… maybe getting psychological help would make a difference…”

## Discussion

**Figure 1:**
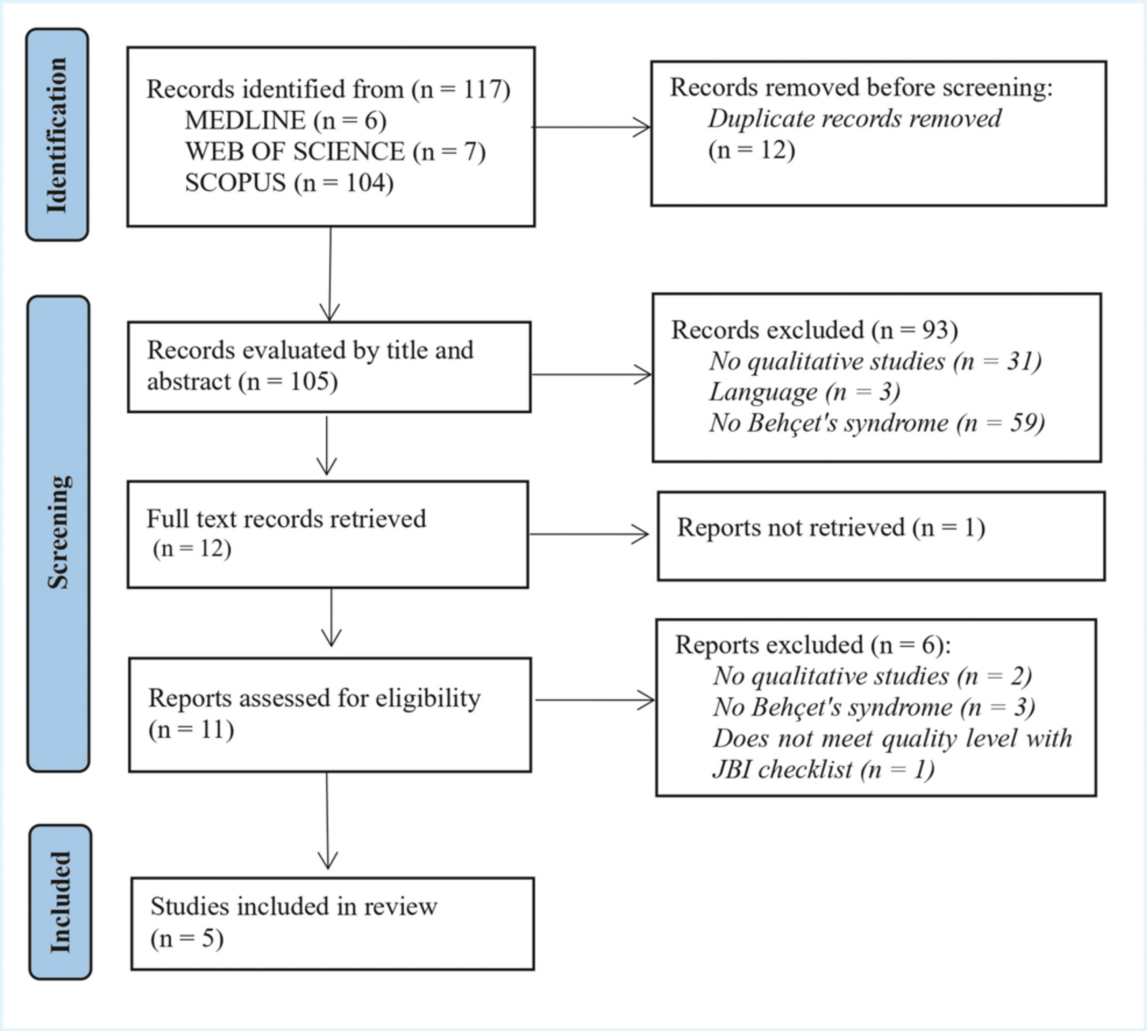
PRISMA flowchart of included studies Image credit: [16] PRISMA, Preferred Reporting Items for Systematic reviews and Meta-Analyses

BS significantly affects quality of life, profoundly impacting physical, emotional, psychological well-being, and social interaction. To corroborate these aspects, prior to the interview, a review of qualitative studies (Phase 1) was conducted to identify relevant areas of interest in the scientific literature. During the review process, a total of 117 records were retrieved. After removing duplicates (n = 12), 105 records were screened by title and abstract, leading to the exclusion of 93 records. The remaining 12 records underwent full-text screening, with six excluded, resulting in five studies included in the review. The Preferred Reporting Items for Systematic Reviews and Meta-Analyses (PRISMA) flowchart in Figure 1 illustrates the selection process of studies included in the review [16].

The critical appraisal revealed that most studies demonstrated good methodological quality. Five of the six studies met between 70% [17] and 100% [18] of the evaluation criteria and were therefore included. The study by Marinello et al. [19] was excluded due to several concerns regarding key quality items and a low compliance score of 30%, as detailed in Table 3, which presents the critical appraisal of the included studies.

**Table 3:** Critical appraisal of the included studies using the Joanna Briggs Institute ckecklist Y*: Yes; N†: No; D‡: Unclear (Q1) Is there congruity between the stated philosophical perspective and the research methodology? (Q2) Is there congruity between the research methodology and the research question or objectives? (Q3) Is there congruity between the research methodology and the methods used to collect data? (Q4) Is there congruity between the research methodology and the representation and analysis of data? (Q5) Is there congruity between the research methodology and the interpretation of results? (Q6) Is there a statement locating the researcher culturally or theoretically? (Q7) Is the influence of the researcher on the research, and vice-versa, addressed? (Q8) Are participants, and their voices, adequately represented? (Q9) Is the research ethical according to current criteria or, for recent studies, and is there evidence of ethical approval by an appropriate body? (Q10) Do the conclusions drawn in the research report flow from the analysis or interpretation of the data?

Author and year	Q1	Q2	Q3	Q4	Q5	Q6	Q7	Q8	Q9	Q10	Score (%)
Jahani et al. (2024) [20]	Y*	Y	Y	Y	Y	N†	N	Y	Y	Y	8/10 (80)
Marinello et al. (2023) [19]	Y	Y	D‡	D	D	N	N	N	Y	D	3/10 (30)
Khaton Taheri et al. (2021) [21]	Y	Y	Y	Y	Y	N	N	Y	Y	Y	8/10 (80)
Sweeting and Arden-Close (2020) [18]	Y	Y	Y	Y	Y	Y	Y	Y	Y	Y	10/10 (100)
Ozguler et al. (2019) [17]	Y	Y	Y	Y	Y	N	N	Y	Y	N	7/10 (70)
Tai et al. (2017) [22]	Y	Y	Y	Y	Y	N	N	Y	Y	Y	8/10 (80)

The included studies were conducted in various countries, reflecting geographic diversity in the qualitative exploration of BS: Turkey [17], the United Kingdom [18], Iran [20,21], and New Zealand [22]. Regarding methodological approach, two studies [18,20] used an interpretative phenomenological analysis, while the remaining studies did not explicitly specify a methodological framework [17,21,22]. All studies employed in-depth, semi-structured interviews for data collection [17,18,20-22], with Khaton Taheri et al. [21] additionally using structured observations, as shown in Table 4, which presents the socio-demographic characteristics, methodological approaches, and data collection techniques of the included studies.

**Table 4:** Socio-demographic characteristics, methodological approaches, and data collection techniques of the included studies

Author and year	Country	Methodological approach	Data collection technique	Duration of the interviews	Number of participants	Age, mean (range)	Duration of disease, mean (range)
Jahani et al. (2024) [20]	Iran	Interpretative phenomenological	In-depth interviews (9 face-to-face and 6 videocalls)	40 minutes	15 (11 women and 4 men)	25-53	2-22
Khaton Taheri et al. (2021) [21]	Iran	Does not specify approach	22 individual interviews and 8 structured observations	45-60 minutes	20 (13 women and 7 men)	50	10
Sweeting and Arden-Close (2020) [18]	UK	Interpretative phenomenological	Semi-structured in-depth interviews (5 video calls, 1 telephone, and 1 face-to-face)	45-90 minutes	7 women	35-40	7.7
Ozguler et al. (2019) [17]	Turkey	Does not specify approach	Semi-structured in-depth interviews and 41 open-ended questions	30-40 minutes	20 (5 women and 15 men)	29-46	11 (9-18)
Tai et al. (2017) [22]	New Zealand	Does not specify approach	Semi-structured in-depth interviews	90 minutes	8 (6 women and 2 men)	51 (24-67)	16.5 (1-32)

Across the included studies, a total of 14 themes and 55 sub-themes were identified. These are detailed in Table 5, which presents the themes and sub-themes extracted from the review.

**Table 5:** Themes and sub-themes identified in the review

Author and year	Themes	Sub-themes
Jahani et al. (2024) [20]	Emotional, mental, and communicative instability	Indecision and uncertainty/Giving up desires and wishes/The feeling of despair and fainting/Fears caused by the results of the disease/Sadness and mental anguish/Isolation
	Psychological and communicative reinforcement strategies	Reluctance to seek support/Having an occupation/Seeking support/Expecting miracles and healing/Doing mental exercises/Engaging in hobbies
Khaton Taheri et al. (2021) [21]	Obstacles to treatment adherence	Denial of illness/Inability to cope with treatment/Concealment of illness/Discontinuation of medication due to adverse effects/Financial burden of treatment/Difficulties in accessing health care/Difficulties in making appointments/Transport barriers
	Facilitators of treatment adherence	Hope for recovery/Incentives/Combining faith with action/Fear of consequences of not following treatment/Family support/Receiving support/Peer support/Interaction with and medical guidance from the treatment team/Acceptance of the disease/Effort to adapt to the disease/Having medication reminder systems in place to overcome forgetfulness/Regular health visits
	Aspects missing from the treatment program	Doubts about treatment efficacy/Inadequate patient education/Discontinuation of treatment after remission/Inadequate knowledge of treatment/Neglect of physical activity/Neglect of lifestyle modification/Lack of balanced diet/Inadequate management of psychological distress
Sweeting and Arden-Close (2020) [18]	Problems due to lack of understanding of symptoms	Misdiagnosis of genital herpes
	Problems due to Behçet's symptoms	Genital manifestations/Painful sex/Exhaustion
	Difficulties in communicating with medical professionals	Feeling unable to raise the problem/Lack of knowledge
	Impact of medication on intimacy	Positive effect/Negative effect
	Partner support	-
	Problems in seeking advice on intimacy from non-health professionals.	Online advice/Suggestions for improvement
Ozguler et al. (2019) [17]	Disease symptoms	Mucocutaneous symptoms/Pain/Eye symptoms/Fatigue and sleep disturbances/Gastrointestinal symptoms
	Functional impact	Impact on speech/Impact on vision/Impact on mobility/Impact on daily activities and work
	Psychological impact	Emotions/Emotional management techniques
	Social impact	Ability to socialize/Impact on family relationship/Impact on work
Tai et al. (2017) [22]	Diagnosis	Diagnostic Challenge/Diagnostic Closure
	Impact of illness	Pain/Fatigue/Reduced vision/Fear and uncertainty
	Loneliness and isolation	Rare disease: Lack of support and information/Invisible disease/Trivialization of the disease
	Acquisition of resilience	Coping with illness/Gaining a sense of control/Support group
	Ongoing interactions with the healthcare system	Specialized care/Primary care/Need for multidisciplinary care/Physician-patient relationship

The emerging sub-themes and themes from the interview were subsequently compared with those identified in the literature review to recognize patterns consistent with findings reported in previous studies. Notably, the themes and sub-themes identified in our interview closely align with those reported in the qualitative literature on BS. The first theme, Coping and Adaptation Strategies, encompasses aspects such as self-care and seeking support. This is consistent with the literature, which emphasizes psychological impact [17], emotional and communicative instability, strategies for psychological and communicative reinforcement [20], the development of resilience [22], and the role of partner support [18]. These elements highlight the need for interventions focused on strengthening patients’ emotional and social resources.

The second theme, Relationship With the Healthcare System, identified in the interview through sub-themes such as medical care and health education, is supported by literature that underscores communication difficulties with healthcare professionals [18,22], challenges in diagnosis [22], treatment programs, and barriers and facilitators to treatment adherence [21]. Moreover, existing studies note the difficulties patients face in seeking guidance on intimacy outside the medical context [18], reinforcing the importance of a multidisciplinary, patient-centered approach to care.

Finally, the theme Living With the Disease, which includes emotional well-being, physical discomfort, and vulnerability, is reflected in the literature through descriptions of symptoms [17], symptom-related problems, lack of symptom understanding, the impact of medication on intimacy [18], and the broader functional and social consequences of the condition [17]. The literature also highlights feelings of isolation [20,22], loneliness, and the burden that the disease imposes on daily life [22], emphasizing the complex reality of living with BS and the need for comprehensive support.

This alignment between the themes emerging from the interview and those reported in the literature not only reinforces the validity of qualitative findings but also highlights critical areas for future clinical and psychosocial interventions, particularly those focused on enhancing communication, emotional support, and overall quality of life for patients [23]. Importantly, patient-provider interaction emerged as a key factor in ensuring comprehensive, human-centered care [24].

Notably, and consistent with previous studies [17,18,20-22], the participant described experiences of delayed diagnosis, lack of effective treatment options, insufficient information, and limited access to both healthcare and social resources for disease management. These issues contributed to a perceived sense of inequality compared to individuals living with more common illnesses. During the diagnostic process, patients often encounter a lack of both information and support, leading to frustration, emotional strain, and significant barriers to accessing appropriate care [25]. Healthcare systems should promote therapies that encourage autonomy - such as physical therapy, rehabilitation, and psychological support - to help manage the emotional and physical burden [26]. However, training of healthcare professionals in rare diseases remains limited, which hinders effective, person-centered care. A holistic, multidisciplinary, and communicative approach is required to provide tailored resources, streamline diagnosis, and ensure access to appropriate care. This is especially relevant in primary care, where initial contact often occurs, and long-term follow-up may be managed [26]. Recent initiatives have aimed to improve healthcare systems’ capacity to address rare diseases through the creation of collaborative networks. At the European level, 24 European Reference Networks (ERNs) bring together specialized centers for multidisciplinary diagnosis and treatment [27].

Regarding the lived experience of BS, symptoms have a notable impact on patient functionality. This was reflected in the present narrative and is consistent with previous studies [17,18,20,22]. Ocular inflammation and joint pain severely impair mobility, affecting work and household activities. Oral ulcers hinder speech, social relationships, and eating, while genital ulcers cause discomfort during urination and sexual activity. Chronic pain and persistent fatigue further intensify symptom burden, drain energy, and hinder daily functioning. Psychological factors, such as anxiety and depression, are closely linked to BS symptoms, with psychological distress aggravating the clinical picture [28]. Fatigue, in particular, has been associated with psychological comorbidities and chronic pain, amplifying the negative impact [29]. As a result, psychosocial deterioration can worsen the clinical course. Therefore, psychological care is essential for patients with rare diseases [28].

These individuals - especially children and young adults - often experience stigma, low self-esteem, difficulty developing coping strategies, and a sense of being different. They may feel socially isolated or discriminated against in school, work, or community settings. For this reason, participatory approaches that facilitate the expression of their experiences and needs are essential to developing individualized care plans [30]. Families are also deeply affected, facing stress and anxiety due to prolonged diagnostic processes, uncertainty, and lack of resources, particularly when emotional support is lacking [25].

Importantly, how patients manage their illness influences both their quality of life and that of those around them. Coping skills are crucial to successful adaptation, as reflected in both the present case and prior research [17,20-22]. It is, therefore, important to promote positive reappraisal strategies, emotional regulation, behavioral adjustment, and self-control to facilitate adaptation to stressful situations and enhance quality of life.

However, studies such as that by Atay and Erturan [31] show that BS patients tend to use fewer of these active coping strategies, instead relying more on passive approaches. Psychiatric disorders such as depression and anxiety are strongly associated with passive coping styles, such as avoidance, self-blame, and disengagement. These maladaptive strategies undermine patients’ ability to manage stress, leading to poorer outcomes [31]. In contrast, active coping styles foster resilience and a better quality of life.

Notably, while confrontational coping is classified as active, it has been identified as the strongest predictor of depression and reduced quality of life in BS patients. This style involves aggressive attempts to change one’s circumstances, often leading to risky behaviors and poor emotional regulation. For this reason, healthcare providers must offer clear, empathetic communication as a foundation for effective therapeutic relationships, and discourage harmful coping responses [31].

Another key factor is the support patients receive [17,18,20-22]. Emotional support strengthens resilience, self-esteem, and coping capacity, while also reducing feelings of isolation. Nevertheless, it is important to note that some programs may deliver inaccurate information or have a negative emotional impact [32]. Therapeutic support - whether virtual or face-to-face - should be tailored to patient needs. In-person settings offer psychosocial benefits, while virtual platforms help overcome access barriers for patients with physical or emotional limitations [33].

Regarding the limitations of the Phase 1 research, the small number of qualitative studies identified limits the ability to obtain broader patterns and information on the experiences of people with BS. Also, some studies could have been excluded because of language limitations (Spanish and English). Regarding Phase 2, the main limitation was the participation of a single informant, which prevented obtaining different perspectives and experiences in BS. Despite the effort of the researchers to maintain rigor and reflexivity during the study, the previous relationship of the interviewer with the participant could have influenced the development of the interview, conditioning the interpretation of the data. Although the researcher conducting the biographical-narrative interview had a prior relationship with the informant, a factor that may introduce bias, this limitation was explicitly acknowledged and addressed through methodological rigor. In particular, two external researchers (CMDG and HGdlT), who were not involved in data collection or initial analysis, independently reviewed and interpreted the data, thereby strengthening the credibility and confirmability of the findings. The decision to include a single participant in the narrative phase was intentional and aligned with the exploratory nature of the study, as well as with the principles of biographical-narrative methodology, which emphasize interpretive depth over generalizability. The objective was not statistical representativeness, but rather a nuanced understanding of an individual’s lived experience with BS, consistent with purposive sampling practices commonly employed in qualitative research. While the prior acquaintance between interviewer and participant could raise concerns regarding neutrality, it also fostered a trusting environment that facilitated the emergence of rich, reflective insights that might not have been elicited otherwise. Reflexivity was actively integrated throughout the process, including the use of a field journal to document decisions and assumptions during analysis. Overall, despite limitations in terms of transferability and potential bias, the study offers exploratory value and interpretive depth, contributing relevant insights to inform both future research and clinical or psychosocial care strategies for individuals living with BS.

As implications for clinical practice, BS requires comprehensive care that includes physical, emotional, and social aspects. Improving the training of professionals, access to multidisciplinary treatments, and strengthening support networks are essential measures that can contribute to the quality of life of patients and their families. Nursing, due to its holistic approach and proximity to the patient, plays a fundamental role in the care of these pathologies, promoting coping and adaptation strategies.

## Conclusions

Qualitative literature on BS is limited, highlighting delayed diagnosis, ineffective treatments, and poor continuity of care, with a clear need for greater psychological and social support - particularly for children and adolescents, who remain under-researched despite the disease's significant impact on their development. The participant’s testimony offered valuable insights into daily challenges, helping to make lived experiences visible and inform personalized interventions. This study offers an in-depth and nuanced view of living with BS through the biographical narrative of a single case, complemented by a broader qualitative synthesis. While the findings are not generalizable, they offer valuable interpretative insights that enhance understanding of the complexity of self-care and adaptation to a rare chronic illness. The integration with existing literature and theoretical frameworks, such as Riegel’s middle-range theory of self-care, adds conceptual depth, although the boundaries between individual experience and broader patient trends are acknowledged as limited. Therefore, further qualitative research is needed to deepen understanding of patient experiences and improve care from a multidisciplinary perspective, optimizing diagnosis, treatment, and access to resources that support quality of life. Caution is advised when extrapolating the results of this study, and the importance of conducting additional studies with a greater diversity of narratives is emphasized to broaden and validate the interpretative scope.
